# Mindfulness video game improves connectivity of the fronto-parietal attentional network in adolescents: A multi-modal imaging study

**DOI:** 10.1038/s41598-019-53393-x

**Published:** 2019-12-10

**Authors:** Elena G. Patsenko, Nagesh Adluru, Rasmus M. Birn, Diane E. Stodola, Tammi R. A. Kral, Reza Farajian, Lisa Flook, Cory A. Burghy, Constance Steinkuehler, Richard J. Davidson

**Affiliations:** 1 Center for Healthy Minds, University of Wisconsin – Madison, 625W. Washington Avenue, Madison, WI 53703 USA; 2 Department of Psychiatry, University of Wisconsin – Madison, 6001 Research Park Blvd., Madison, WI 53719 USA; 3 Department of Psychology, University of Wisconsin – Madison, 1202 West Johnson Street, Madison, WI 53706 USA; 4 Department of Informatics, University of California, Irvine, 5019 Donald Bren Hall, Irvine, CA 92697-3440 USA

**Keywords:** Attention, Human behaviour

## Abstract

Mindfulness training has been shown to improve attention and change the underlying brain substrates in adults. Most mindfulness training programs involve a myriad of techniques, and it is difficult to attribute changes to any particular aspect of the program. Here, we created a video game, Tenacity, which models a specific mindfulness technique – focused attention on one’s breathing – and assessed its potential to train an attentional network in adolescents. A combined analysis of resting state functional connectivity (rs-FC) and diffusion tensor imaging (DTI) yielded convergent results – change in communication within the left fronto-parietal network after two weeks of playing Tenacity compared to a control game. Rs-FC analysis showed greater connectivity between left dorsolateral prefrontal cortex (dlPFC) and left inferior parietal cortex (IPC) in the Tenacity group. Importantly, changes in left dlPFC – IPC rs-FC and changes in structural connectivity of the white matter tract that connects these regions –left superior longitudinal fasiculus (SLF) – were associated with changes in performance on an attention task. Finally, changes in left dlPFC – IPC rs-FC correlated with the change in left SLF structural connectivity as measured by fractional anisotropy (FA) in the Tenacity group only.

## Introduction

Video gaming is prevalent in the modern world. In the US alone, 134 million people (65% of the population) play video games regularly - three hours of more per week^1^. Among adolescents, the number is even higher – 97% of American teenagers (age 12 to 17 years) play video games on a regular basis^2^. Although video games are typically developed and used for entertainment purposes, there has been a notable interest in their educational potential. Research indicates that video games can facilitate acquisition of declarative knowledge (e.g. biological concepts in Virulent^3^); or train cognitive processes (e.g. spatial imagery in Tetris^4^; visual processes in Unreal Tournament^5^), see^6^ for review.

At the same time there is a growing recognition of the potentially deleterious effects of video games, including problems in attention, particularly in adolescents^7^. It is especially in light of these data that we wished to explore whether we could develop a video game incorporating simple mindfulness practices that might actually improve attention. Here we developed a video game - Tenacity - that incorporates a simple mindfulness meditation technique into its game mechanics and then we assessed the game’s impact on attention in adolescents. We used a randomized controlled trial design with behavioral measure of attention and functional and structural measures of brain circuits underlying attention as outcome measures.

A contemporary framework groups mindfulness meditation techniques into two broad categories: focused attention and open monitoring or open awareness^8^. Focused attention meditation involves sustained attention on an object or bodily sensation, often one’s breathing. If attention drifts away from the chosen object, the meditator practices disengaging from the distractor and re-focusing attention on the object. Open awareness meditation, on the other hand, involves attention to the environment as a whole - both internal and external - without focusing on a particular object, and without losing meta-awareness of the entire process. If meta-awareness is lost by being captured by a thought or an object, the meditator practices returning to the state of monitoring the environment and re-establishing meta-awareness.

**Figure 1:**
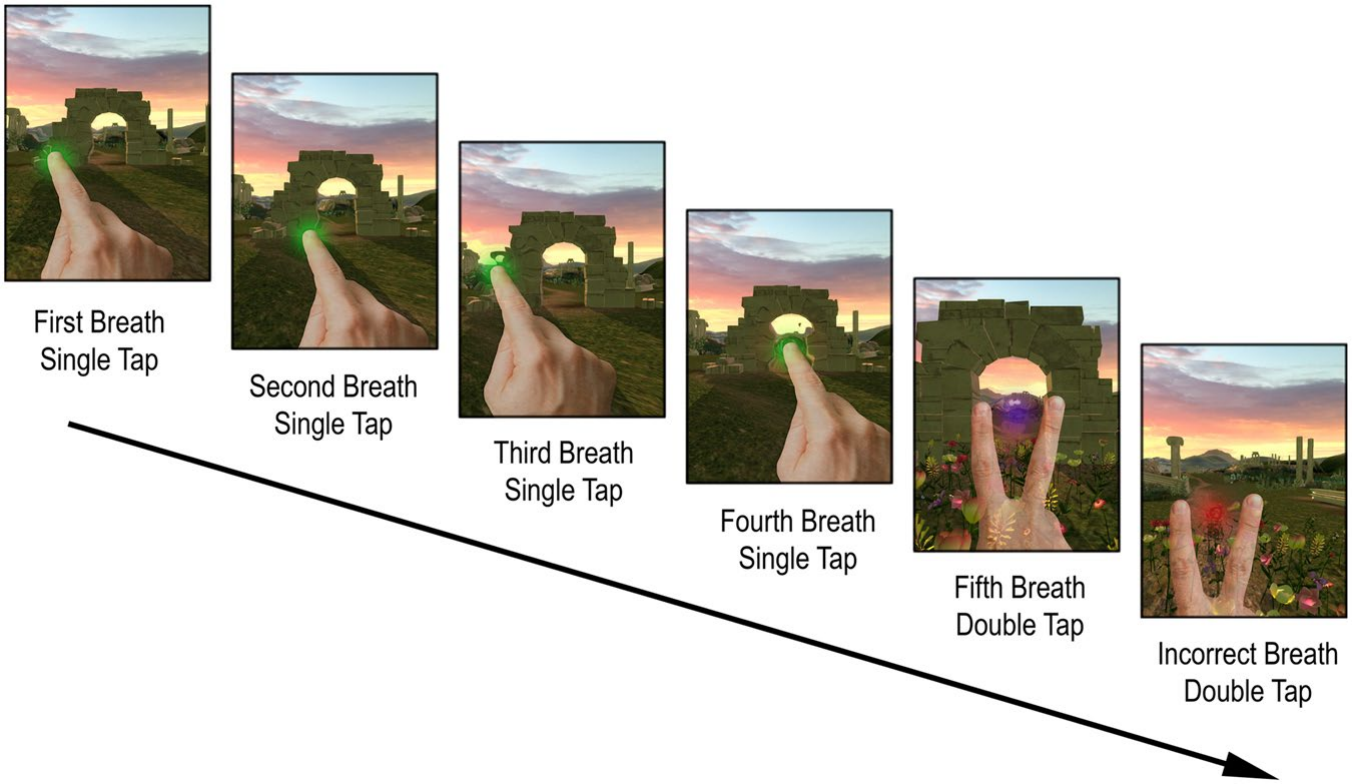
Example timeline for Tenacity game: Players must tap with one finger on the first four outbreath and with two fingers on the fifth outbreath. Here, the first five images demonstrate correct taps, and the last image demonstrates an incorrect tap: a tap with two fingers on the first outbreath of a new cycle.

Tenacity, which was designed after a laboratory task^9^, affords players the opportunity to train focused attention by monitoring the breath (Fig. 1). Specifically, the game involves tapping on an iPad screen with each out-breath. Players must tap with one finger on the first four out-breaths, and with two fingers on the fifth out-breath. Once a five-breath cycle is complete, a new five-breath cycle begins.

Longitudinal^10–15^, and cross-sectional studies^16^ of meditation training, have shown that meditation can improve various aspects of attention and change the underlying brain substrates in adults (see^17^ for review). As few as five days of meditation practice produced changes in an executive component of attention. Following the practice, participants were more efficient in choosing an appropriate response among conflicting alternatives^10^. Importantly, the short-term practice induced an increase in white-matter tract integrity in the anterior and superior corona radiata, the neural substrates that are usually associated with the executive component of attention^11,12^. An EEG study showed that intensive training in focused attention meditation improved selective attention – the ability to detect a target tone among distractors – as evident by a reduction in reaction time variability and an increase in trial-to-trial consistency of the event-related neural response to the task^13^. However, a behavioral study employing a visual discrimination task found no effects of meditation training on sustained attention, but showed an improvement in vigilance^14^. Several functional MRI studies revealed greater activation of dorsolateral prefrontal cortex (dlPFC) – a key area of an attentional network – during executive task performance in meditators compared to an active control group^15,16^. Another fMRI study showed that the practice-related change in activation of the attentional network follows an inverted u-shaped curve. Initially, meditation training leads to an increase in activation; however, after extensive practice (44,000 hours on average) meditation training results in the decreased activation in attention-related brain regions^16^. This phenomenon was interpreted by the authors as reflecting the effortless quality of attention that emerges following long-term practice. The above-described training studies have provided evidence that mindfulness can improve attention; however, a coherent understanding of the mechanism of such improvement is still missing. We attribute the diversity of the results to that fact that previous research on meditation employed highly complex training regimes that varied from study to study (e.g. MBSR, IBM). The advantage of the current approach is that we modeled one particular aspect of mindfulness training in Tenacity – attention to one’s breathing. Thus any effects that we found in the present study can be attributed to this particular technique.

Research reveals neural plasticity underlying the successful training of attentional networks throughout the lifespan^11,13,18^. Adolescence could be a critical period for such training since it is characterized by a steeper change in executive functions – improvements in reaction time and accuracy on attentional tasks – compared to adulthood^19–21^. These cognitive changes are supported by protracted maturation of white matter, in particular tracts that involve the frontal lobe – superior longitudinal fasciculus (SLF), uncinate fasciculus, and others^22^. A number of fMRI studies showed age-related differences in recruitment of fronto-parietal regions whereby adolescents fail to recruit inferior parietal cortex on attentional tasks^23–25^. Greater recruitment of parietal regions is positively associated with performance on the executive tasks^26^. A computational simulation revealed that increased engagement of parietal regions development is due to greater connectivity between fronto-parietal regions rather than greater connectivity within the regions themselves^27^. The finding is corroborated by rs-FC studies showing pruning of local connectivity and strengthening of long-range connectivity with age^28^.

In the current study, we used rs-FC and DTI to assess the impact of a meditation-based video game – Tenacity – to train attentional networks. In particular, we hypothesized that playing Tenacity for two weeks would increase left fronto-parietal resting state connectivity and white matter integrity of the tract that connects frontal and parietal regions – left SLF. We also expected that the changes in rs-FC and DTI indices would be associated with improvement on an executive component of attentional task administered outside the scanner. The executive component of attention means here the ability to efficiently choose an appropriate response among conflicting alternatives (i.e. word naming and emotion recognition). We focused on the left dlPFC in particular, because meta-analyses reported the involvement of the left dlPFC in the attentional tasks similar to the one we employed in the current study^29,30^. To test these hypotheses, we randomized adolescents to two weeks of training with either the intervention game, Tenacity, or with the active control game, FruitNinja, for 30 minutes per day (SI Table S1). We chose the commercially available game FruitNinja as a control game because it also involves operating a touch screen, and it is an attentionally demanding game. Before and after training, we acquired rs-FC and DTI data from each participant.

We also administrated an attentional Stroop-like task – the Emotional Conflict task (ECT)^31^. In this task, the participants are presented with the words “HAPPY” or “FEAR” overlaid on faces expressing happy and fearful emotions. Trials were divided evenly into two types. The meaning of the word matched the facial expression on congruent trials, and differed on incongruent trials. The task is to identify the facial expression and ignore the words. Incongruent trials are usually slower and less accurate, because the system needs to resolve the conflict between word naming and facial emotion recognition processes. We chose the emotional version of the Stroop task because it engages similar brain areas as the traditional Stroop task (e.g. left dlPFC, dACC) while the emotional stimuli may create a more intense conflict and potentially improve our chances to detect a behavioral change after a short mindfulness training^29^. Emotional stimuli have been shown to be more attention-capturing than neutral stimuli in a number of paradigms beside the Stroop task^32^, such as a visual search task^9^ and attentional blink task^33^. Recent reviews on emotion-cognition interaction suggest that prefrontal cortex supports a domain-general control mechanism that operates both on emotional and neutral information alike^34,35^.

Our overarching hypothesis was that playing Tenacity would improve performance on the ECT and would alter corresponding functional and structural brain networks known to be involved in attention, compared with playing the commercially available control game.

## Results

This study had four primary aims: (i) to examine changes in adolescent performance on attentional task (ECT) following two weeks of gameplay, (ii) to examine the relation between changes in the left dlPFC rs-FC and changes in adolescent performance on the ECT, (iii) to examine changes in white matter integrity in specific fiber tracts as measured by DTI and changes in the performance on the ECT, and (iv) to examine the relation between the changes in the left dlPFC rs-FC and the changes in white matter integrity. The left dlPFC seed region [−42, 16, 28] was chosen from a meta-analysis performed on 47 neuroimaging studies involving conflict resolution^30^. The region was one of the largest clusters reported for Stroop-like tasks (similar to the ECT). The dlPFC is also a critical region that has been found to change functionally in response to focused attention meditation^16^.

### Emotional conflict task (ECT)

#### Gameplay effects

Two-way repeated measures ANOVAs with trial type (congruent vs incongruent) as within-subject factors and group (Tenacity vs FruitNinja), as a between-subject factor revealed no significant two-way interactions for change in accuracy (Time2 - Time1) *F*(1,85) = 0.132, p = 0.72 and change in reaction time (Time2 - Time1), *F*(1,86) = 0.663, *p* = 0.42 (Table S2) There were also no group effects for change in accuracy *F*(1,85) = 3.476, *p* = 0.07 and change in reaction time *F*(1,86) = 3.727, *p* = 0.06.

#### Congruency effects

The main effect for congruency was significant, change in accuracy: *F*(1,85) = 8.49, *p* = 0.0046, change in reaction time: *F*(1,86) = 23.098, *p* = 0.000066, with congruent trials getting less accurate; and incongruent trials getting faster. No change in RTs on congruent trials could be explained by the ceiling effect – fast RTs at Time1.

Including Gender (Male vs Female) as a between subject variable in the model did not change the pattern of the results; there were no statistically significant Gender effects. No group effects at Time1 were significant.

### Resting state connectivity and ECT

**Figure 2:**
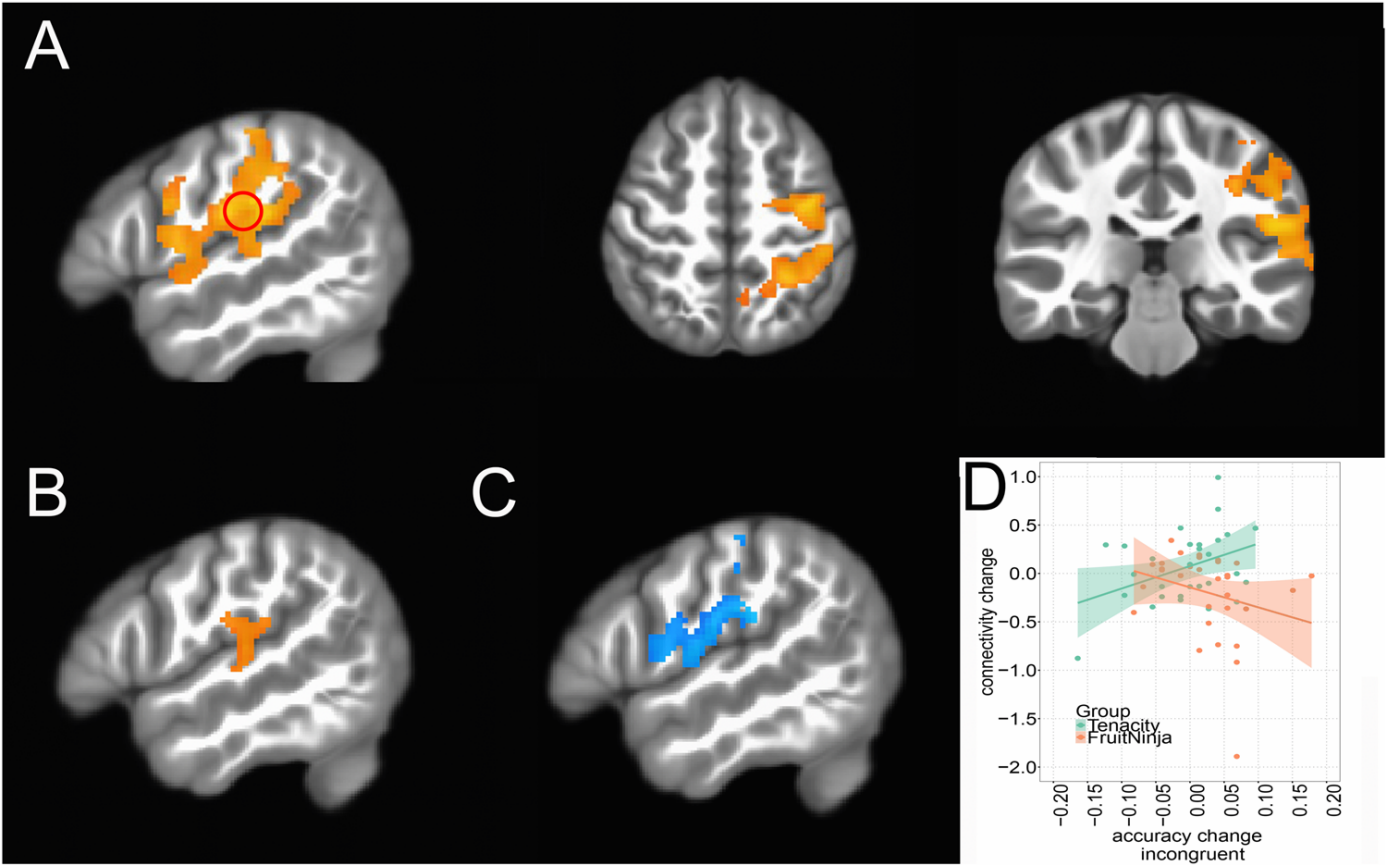
Playing Tenacity for two weeks increased rs-FC between L dlPFC and L IPC (Fisher’s Z). (**A**) A cluster in L IPC showing a group effect in the change in L dlPFC-L IPC rs-FC (*P* < 0.05, whole brain corrected); the red circle represents an independent left IPC ROI [−50, −20, 21] from^36^, chosen to examine relationship of rs-FC and behavior. (**B**) Tenacity group: increase in L dlPFC-L IPC rs-FC from Time1 to Time2 (*P* < 0.05, not corrected). (**C**) FruitNinja group: decrease in L dlPFC-L IPC rs-FC from Time1 to Time2 (*P* < 0.05, not corrected). (**D**) Correlation between change in L dlPFC - L IPC rs-FC and change in accuracy on incongruent trials in the ECT task (*n* = 61).

Whole brain left dlPFC-seeded rs-FC analysis revealed a significant group effect (Tenacity vs FruitNinja) in the change in connectivity (Time2 - Time1) between the left dlPFC and the left IPC (Fig. 2A). The group effect was driven by an increase in rs-FC in Tenacity group (Fig. 2B) and a decrease in rs-FC in FruitNinja group (Fig. 2C). We detected no other significant cluster showing a group effect in the change in connectivity. We also did not find any significant clusters using the right dlPFC as a seed for the rs-FC analysis, suggesting that the observed training effect was specific to the left hemisphere.

Next, to understand the behavioral significance of the change in left fronto-parietal network connectivity, group differences in the relationship between rs-FC and accuracy on incongruent trials, a measure that reflects focused attention, were examined. ROI analysis was performed on an independently defined region in the left IPC (a red circle in Fig. 2A), see SI for more details. The effect of change in rs-FC (Time2 - Time1) on change in accuracy differed by group, slope = −4.36, se = 1.85, 95% CI = [−8.07, −0.66], *t*(61) = −2.356, *p* = 0.02, (one outlier in rs-FC was removed and two outliers in accuracy on incongruent trials were removed). The change in accuracy on incongruent trials was marginally positively correlated with the change in rs-FC in the Tenacity group, slope = 2.32, se = 1.03, 95% CI = [0.21, 4.43] *t*(29) = 2.26, *p* = 0.03; the change in accuracy on incongruent trials did not correlated with the change in rs-FC in the FruitNinja group, slope = −2.04, se = 1.55, 95% CI = [−5.20, 1.12], *t*(31) = −1.32, *p* = 0.2 (Fig. 2D).

### Diffusion tensor imaging and ECT

**Figure 3:**
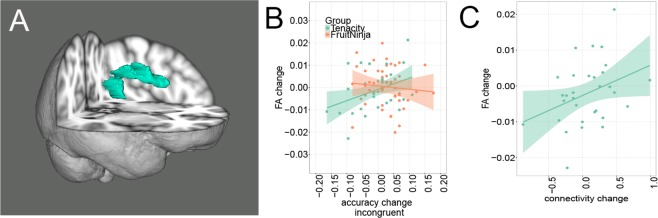
Increase in white matter integrity in L SLF after two weeks playing Tenacity is associated with improvement in accuracy on ECT task. (**A**) L SLF John Hopkins University Atlas (**B**) Group difference in correlation between the change in fractional anisotropy (FA) of L SLF and the change in accuracy on incongruent trials in the ECT task (*n* = 80). (**C**) marginally significant correlation between the change in FA of L SLF and the change in L dlPFC- IPC rs-FC in Tenacity group (*n* = 31).

Having identified changes in functional connectivity between left dlPFC and left IPC after two weeks of gameplay, we examined the possible structural changes in integrity of the white matter tract that connects these two regions – left superior longitudinal fasciculus (L SLF) (Fig. 3A). In particular, we examined fractional anisotropy (FA), which is thought to reflect white matter integrity^36^. We did not find a significant group effect on change (Time2 - Time1) in L SLF FA, *t*(81) = −0.63, *p* = 0.53. However, we found significant group differences in the relationship between the change in FA (Time2 - Time1) and the change in accuracy on incongruent trials, the same trial type that related to increased L dlPFC – IPC rs-FC in the Tenacity group, slope = −0.07, se = 0.03, 95% CI = [−0.13, 0], *t*(80) = −2.015, *p* = 0.047 (one outlier in DTI was removed and two outliers in accuracy on incongruent trials were removed), (Fig. 3B) The change in FA of L SLF positively correlated to the change in accuracy on incongruent trials only in Tenacity group, slope = 0.05, se = 0.02, 95% CI = [0.01, 0.09], *t*(36) = 2.393, *p* = 0.02, the slope was not significant for FruitNinja group, slope = −0.01, se = 0.02, 95% CI = [−0.06, 0.03], *t*(43) = −0.614, *p* = 0.54.

Next, we examined whether the increase in rs-FC between L dlPFC and L IPC that we found in the Tenacity group positively correlated with the increase in L SLF FA (Fig. 3C). Results showed that the change in L dlPFC – L IPC rs-FC (Time2 - Time1) marginally correlated with the change in L SLF FA (Time2 - Time1) in the Tenacity group, slope = 0.01, se = 0.004, 95% CI = [0, 0.02], *t*(30) = 2.013, *p* = 0.054 (one outlier in DTI was removed and one outlier in rs-FC was removed).

## Discussion

The present study examined the effects of video-game-based mindfulness training in an adolescent population using a longitudinal, randomized design. The results show that two weeks of playing a meditation-based video game, Tenacity, can produce significant changes in the fronto-parietal attentional control brain network. Rs-FC analysis showed a greater increase in connectivity between left dlPFC and left IPC in the Tenacity group compared to the control group after two weeks of game-play. Importantly, changes in the left dlPFC – IPC rs-FC and changes in fractional anisotropy of the white matter tract that connects these regions – left SLF – were associated with improvements in the performance on an attentional task. Finally, the change in left dlPFC – IPC rs-FC correlated with the change in fractional anisotropy of left SLF in Tenacity group only.

The developmental significance of improved connectivity between L dlPFC and L IPC is highlighted by a number of fMRI studies that show that adolescents normally fail to recruit the left IPC while performing attentional tasks^23–25^. In a task-switching paradigm, where participants were asked to respond either to the color or to the direction of a moving target, younger participants (8–13 years old) were slower and less accurate compared to adults^24^. fMRI results of that study showed that left IPC was among few regions that was significantly less active in adolescents than in adults across all trial types. In a stimulus-response compatibility paradigm, left IPC was the only area that showed a group effect between children aged 8–11 and older adolescents aged 14 and 15 years across all trial types^37^. The involvement of left IPC specifically on incongruent trials was revealed in another fMRI study^25^: adults activated IPC bilaterally on incongruent versus neutral trials in an Eriksen flanker task, while adolescents recruited only right IPC. Thus, the increased left dlPFC-IPC connectivity after playing Tenacity for two weeks suggests that the game can potentially aid adolescents in developing a mature pattern of brain activation on attention-demanding tasks.

While we found the group effect in the changes in fronto-parietal connectivity that has been associated in previous literature with attentionally demanding tasks, we did not find a group effect in changes in the behavioral indices of attention (i.e. accuracy or RTs on incongruent trials). This may be due to the possibility that the ECT task is powered for individual difference analyses rather than group effects that benefit from reduced between subject variability. Another important limitation of the study is that a significant number of participants was excluded from rs-MRI analyses for excessive motion and claustrophobia. We believe it is an inherent challenge when scanning participants of this age group (11–13 years old children). For most of them, it was the first time in the scanner, so they had no way of knowing and reporting their claustrophobia during the screening period.

Most previous research on meditation involves highly complex training regimes making it difficult to isolate the active ingredients of change. Mindfulness programs usually include a myriad of components: cultivating non-attachment, equanimity, compassion, attention to one’s posture and breathing, awareness of one’s thoughts to name a few. While the advantage of the current study is that we modeled one particular aspect and type of mindfulness training in Tenacity – attention to one’s breathing. The current design still cannot fully speak to the specificity of this technique on brain change. Other aspects of mindfulness (e.g. nonjudgmental attitude) were not directly compared.

A common challenge for any type of training is that people stop following the program once the class is over. The fact that video gaming is so prevalent among adolescents gives us hope that Tenacity and similar games can serve as a scaffold to support continuous practice in this population.

## Materials and Methods

### Participants

Ninety-five healthy adolescents were recruited from the Madison, WI community and randomly assigned to a Tenacity group or a FruitNinja group, see SI Table S1 for detailed demographic information. The University of Wisconsin-Madison Health Sciences Institutional Review Board approved all study procedures, which were carried out in accordance with the approved guidelines. All participants provided informed assent and were given monetary compensation for their participation. Legal guardians provided written informed consent. The data was collected at the Waisman Center, UW-Madison, Madison, WI.

### Video game intervention

Tenacity game involves tapping on an iPad screen with each out-breath. Players must tap with one finger on the first four out-breaths, and with two fingers on the fifth out-breath. Once a five-breath cycle is complete, a new five-breath cycle begins. In Tenacity, there are two main objectives. The first objective is to synchronize one’s tapping with the natural breathing rate on the iPad screen. The second objective is to count breathing in five-breath cycles by keeping track of where one is in the five-breath cycle. The player receives points and bonuses for every accurately completed five-breath cycle. The player also receives immediate feedback on their performance. Green ripples expand from the location of the tap for a correct response, and red for incorrect; and a specific auditory tone changes based on accuracy. At the beginning of the game, a player can choose from multiple immersive environments for the background of the screen – moving forward along a path among ancient ruins, or moving upward on a staircase in space. The motion of the tapping, the visual perception of the ripples, and the auditory perception of the sounds are all presumed to facilitate keeping attention focused on one’s breathing, which normally would be supported almost solely by proprioceptive information from respiratory organs. The control game, FruitNinja, also involves operating a touch screen, and it is an attentionally demanding game: players’ goal is to slice fruits falling from the top of the screen with a finger movement, while avoiding slicing an occasionally presented bomb. Participants were asked to play the games for 30 minutes every day over two-week period; no one was excluded from the analyses for failing to meet this criterion, see SI Fig. S3 for details.

#### Emotional conflict task

Stimuli were presented with E-prime 2.0 software on a desktop computer screen. The task consisted of photographs of happy and fearful faces with words HAPPY and FEAR written on top of the photographs in red ink, see^31^ for details. The stimuli were presented sequentially for 1,000 ms each, and a fixation cross was displayed between the stimuli for a variable duration (ISI = 3,000; 4,000; or 5,000 ms). There was an equal number of congruent and incongruent trials, totaling 148 trails.

### Rs-FC: data acquisition

MRI data was acquired on a General Electric 3 T MR750 MRI scanner (Waukesha, WI). Resting-state functional MRI data were acquired with a series of sagittal T2*-weighted echo-planar images using a 32-channel receive-only RF-coil (Nova Medical, Wilmington, MA). (TR: 2000ms, TE: 20 ms, matrix: 64 × 64, FOV: 22 cm, 36 slices, slice thickness: 4.0 mm/0.5 mm gap, Flip Angle: 60 degrees, 264 time points). T1-weighted structural data were acquired using the MPnRAGE sequence, which is an inversion recovery prepared, fast gradient echo sequence with three-dimensional (3D) radial k-space sampling as described in^38^. Specific MPnRAGE acquisition parameters were: spatial resolution = 1.0 mm × 1.0 mm × 1.0 mm, whole head coverage (sagittal scans used non-selective RF excitation), TR = 4.6 ms, TE = 1.7 ms, nominal flip angle 4 degrees. See SI for more details. Data analysis: Functional MRI resting-state data analyses were performed using AFNI^39^ analysis package, unless otherwise indicated. See SI for more details.

### DTI: data acquisition

Multi-shell diffusion weighted imaging (DWI) data were acquired from three healthy teenage volunteers on a GE 3.0 T scanner with b-values of 350, 800, and 2500 s·mm^-2^ and respective encoding directions per shell of 9, 18 and 35. In addition, six non-diffusion weighted (b = 0) volumes were also acquired. The voxel resolution was set at 2 × 2 × 2 mm^3^ with the matrix size of 128 × 128 in plane and 72 slices. Image pre-processing: Brain tissue mask was extracted from a b = 0 image using brain extraction tool of FSL^40^. The distortions introduced by eddy currents are corrected using a Gaussian process model based correction implemented in the ‘eddy’ tool of FSL^41^. A multi-compartment tissue model named neurite orientation dispersion and density imaging (NODDI) was fit to the corrected DWI signal for each voxel in the brain using a three stage (grid search, gradient descent and Markov Chain Monte Carlo) fitting procedure^42^. The intrinsic parallel diffusivity was set to 1.7 × 10^−9^ m^2^·s^−1^ in the estimation procedure. From the estimated model extra-cellular diffusion tensors are reconstructed allowing us to extract the traditional diffusion tensor image (DTI) measures such as the fractional anisotropy (FA) and mean diffusivity (MD). The NODDI model itself offers neurite density, orientation dispersion and free-water fraction maps. DTI Unbiased study specific coordinate system, see SI. DTI ROI analysis is described in SI^43–54^.

## Supplementary information

Supplementary Information

## Data Availability

The datasets generated and analyzed during the current study are available from Dr. Richard J. Davidson (rjdavids@wisc.edu) on reasonable request.
