# Supplementary material for: Mindfulness video game improves connectivity of the fronto-parietal attentional network in adolescents: A multi-modal imaging study
Source: Sci Rep. 2019 Dec 10;9:18667. doi: 10.1038/s41598-019-53393-x (PMC6904443; doi:10.1038/s41598-019-53393-x)
Supplement: Supplementary file 1 — Supplementary Information [file 41598_2019_53393_MOESM1_ESM.pdf]

Title

Mindfulness video game improves connectivity of the fronto-parietal attentional network in adolescents: A multi-modal imaging study

Authors:

Elena G Patsenko*[1,5] , Nagesh Adluru[1], Rasmus M Birn[2], Diane E Stodola[1], Tammi RA Kral[1,3], Reza Farajian[1], Lisa Flook[1], Cory A Burghy[1], Constance Steinkuehler[4], Richard J Davidson[1,3]

1. Center for Healthy Minds, University of Wisconsin – Madison, 625 W. Washington Avenue, Madison, WI 53703

2. Department of Psychiatry, University of Wisconsin – Madison, 6001 Research Park Blvd., Madison, WI 53719

3. Department of Psychology, University of Wisconsin – Madison, 1202 West Johnson Street, Madison, WI, USA 53706

4. Department of Informatics, University of California, Irvine, 5019 Donald Bren Hall, Irvine, CA 92697-3440

5. *Correspondence: patsenka@wisc.edu (608) 556-1728

Supplementary Information

Table S1. Demographic characteristics.

Characteristics	Tenacity (N=47)	FruitNinja (N=48)
Age (years)	12.8 (0.7)	12.7 (0.7)
Gender		
Female	16	15
Male	31	33
SES		
$15,000 or less	3	0
$15,001 - $20,000	1	1
$20,001 - $30,000	0	2
$30,001 - $40,000	4	1
$40,001 - $50,000	4	4
$50,001 - $60,000	1	2
$60,001 - $70,000	1	2
$70,001 - $80,000	6	3
$80,001 - $90,000	2	0
$90,001 - $100,000	3	4
$100,001 - $150,000	15	16
$150,001 - $200,000	5	5
> $200,000	3	5
Household size	4.18 (1.09)	4.00 (1.06)

Ethnicity/Race		
African-American	3	3
Asian	2	1
Hispanic	3	2
White	39	39
Does not wish to provide the info	0	2

Footnote: The groups were matched on video game experience ($\chi 2$ = 0.51, p=0.92); and on iPad usage ($\chi 2$ = 1.36, p=0.85).

BEHAVIORAL DATA

Emotional conflict task

Table S2. Change in Accuracy and RTs (Time 2 – Time1) means and standard deviations on Emotional Conflict task.

	Accuracy		RT (ms)	
	Congruent	Incongruent	Congruent	Incongruent
Tenacity	-.02 (.06)	.00 (.07)	21 (59)	-1 (62)
FruitNinja	-.01 (.03)	.02 (.05)	0 (68)	-32 (59)

Materials and Methods

Participants: Ninety-five healthy adolescents were recruited from the Madison, WI community and randomly assigned to a Tenacity group or a FruitNinja group. See SI Table S1 for detailed demographic information. The study was approved by UW-Madison's Health Sciences

Institutional Review Board, and all participants provided informed consent and were given monetary compensation for their participation.

Emotional Conflict Task:

Participants: One participant withdrew prior to Time 2 data collection. Three participants were excluded from ECT analyses due to low accuracy on the task (two participants had 3% accuracy on incongruent trials at Time 1; one participant had 14% accuracy on incongruent trials at Time 1 and 8% accuracy on congruent trials as Time 2); the participants did not seem to understand or follow the instructions. The outliers defined as 3 standard deviations from the mean were excluded from the analyses (four participants met that criterion for RTs analysis and four participants – for Accuracy analysis). To assess the training effects, the difference scores, Time 2 – Time 1 were calculated. To examine the intervention effects, two-way ANOVAs were performed on the difference scores using R statistical language (R Core Team, 2013).

Stimuli were presented with E-prime 2.0 software on a desktop computer screen. The task consisted of photographs of happy and fearful faces with words HAPPY and FEAR written on top of the photographs in red ink, see [1] for details. The stimuli were presented sequentially for 1,000 ms each, and a fixation cross was displayed between the stimuli for a variable duration (ISI =3,000; 4,000; or 5,000 ms). There were 148 trials, divided equally between congruent and incongruent trials.

Brain Imaging data acquisition:

MRI data was acquired on a General Electric 3T MR750 MRI scanner (Waukesha, WI). Resting-state functional MRI data was acquired with a series of sagittal T2*-weighted echo-planar images using a 32-channel receive-only RF-coil (Nova Medical, Wilmington, MA). (TR: 2000ms, TE: 20ms, matrix: 64x64, FOV: 22cm, 36 slices, slice thickness: 4.0mm/0.5mm gap, Flip Angle: 60 degrees, 264 time points). T1-weighted structural data were acquired using the

MPnRAGE sequence, which is an inversion recovery prepared, fast gradient echo sequence with three-dimensional (3D) radial k-space sampling [2]. Specific MPnRAGE acquisition parameters were: spatial resolution = 1.0 mm x 1.0 mm x 1.0 mm, whole head coverage (sagittal scans used non-selective RF excitation), TR = 4.6 ms, TE = 1.7 ms, nominal flip angle 4 degrees. Data acquisition began within 10 ms after the midpoint of the preparation pulse and occurred for approximately 1500 ms. The delay time T_D between the last RF excitation of each readout block and the next preparation pulse was approximately 500 ms. A multi-echo gradient sequence was used in conjunction with the Iterative Decomposition of Water and Fat With Echo Asymmetry and Least-Squares Estimation (IDEAL) algorithm to produce B0 maps.

Resting-state data analysis

Participants: One participant withdrew prior to Time 2 data collection. Twenty-seven participants' data were unusable due to technical issues (five participants), logistical issue (one participant), claustrophobia (three participants), abnormal anatomy (one participant), excessive motion at either one of the two scans (sixteen participants), falling asleep (one participant). The outliers defined as 3 standard deviations from the mean were excluded from the analyses (one participant met that criterion).

Functional MRI resting-state data analyses were performed using AFNI [3] analysis package, unless otherwise indicated. Reconstructed echo-planar image volumes were first corrected for motion using rigid-body realignment (3dvolreg) and corrected for slice-timing differences (3Tshift). The first 3 images (6s) were removed to allow magnetization to reach equilibrium. Data were then corrected for B0-field distortions using customized in-house software that calls the FMRIB Software Library, FSL [4] functions PRELUDE and FUGUE. Images were then aligned to the T1-weighted structural image using an affine transformation and a local Pearson correlation cost function [5]. T1-weighted structural data were aligned to the MNI template using nonlinear warping with ANTS [6]. This warp was then applied to the

preprocessed fMRI data, and resampled to 2mm isotropic resolution. Automated segmentation (FSL's FAST) of the T1-weighted structural image was used to define masks of the WM and CSF [4, 7, 8].

The two signal intensity time-courses resulting from averaging the fMRI data within the eroded WM and CSF masks and their first derivatives (computed by backwards difference) were taken as signals of no-interest (i.e., spurious fluctuations unlikely to be of neuronal origin) and removed from the functional data along with the six rigid-body motion registration parameters [9, 10]. Time points where the sum-squared difference (ssd) of consecutive points of the 6 motion realignment parameters exceeded 0.25 mm were censored and ignored in this nuisance regression. The functional images were temporally band-pass filtered between 0.01 Hz and 0.1 Hz, and spatially smoothed with a 3-dimensional Gaussian kernel (FWHM = 6 mm).

Functional connectivity was computed using a seed-to-voxel connectivity approach [11]. The left dlPFC seed region of interest [-42, 16, 28] was defined from an fMRI meta-analysis performed on 47 neuroimaging studies involving conflict resolution [12]. The region was one of the largest clusters reported for Stroop-like tasks (similar to the ECT). The dlPFC is also a critical region that has been found to change functionally in response to focused attention meditation [13]. The preprocessed fMRI data were averaged over the seed region of interest, and then regressed against all voxels in the brain. Time points with excessive motion (ssd > 0.25 mm) were censored. Functional connectivity maps were corrected for multiple comparisons using a cluster-threshold approach. The spatial smoothness of the preprocessed fMRI data was estimated using AFNI's 3dFWHMx [3]. The resulting estimated FWHM was used in a Monte Carlo simulation (3dClustSim) estimating the likelihood of obtaining clusters of a certain size at various individual voxel p-value thresholds. Using this method, a cluster-corrected size of ≥ 2529 voxels was identified as significant at individual voxel p-value threshold of $p < 0.05$. We estimated the change in functional connectivity by subtracting the functional connectivity

estimates at time point one from the estimates at time point two. To examine the relation between the changes in functional connectivity and changes in behavioral variables, ROI analysis was performed on an independently defined region [14]: a peak connectivity value for a 10 mm sphere was calculated for each participant at each time point. Then the difference between Time 2 and Time 1 was calculated and entered into the linear regression analysis.

Diffusion imaging measures

Participants: One participant withdrew prior to Time 2 data collection. Nine participants' data were unusable due to technical issues (four participants), logistical issue (one participant), claustrophobia (three participants), falling asleep (one participant). The outliers defined as 3 standard deviations from the mean were excluded from the analyses (one participant met that criterion).

Data acquisition

Multi-shell diffusion weighted imaging (DWI) data were acquired on a GE 3.0 T scanner with b-values of 350, 800, and 2500 $s \cdot mm^{-2}$ and respective encoding directions per shell of 9, 18 and 35. In addition, six non-diffusion weighted (b=0) volumes were also acquired. The voxel resolution was set at 2 x 2 x 2 mm^3 with the matrix size of 128 x 128 in plane and 72 slices.

Image pre-processing

Brain tissue masks were extracted from b=0 images using the brain extraction tool of FSL [4]. The distortions introduced by eddy currents were corrected using a Gaussian process model based correction implemented in the 'eddy' tool of FSL [15]. A multi-compartment tissue model named neurite orientation dispersion and density imaging (NODDI) was fit to the corrected DWI signal for each voxel in the brain using a three stage (grid search, gradient descent and Markov Chain Monte Carlo) fitting procedure [16]. The intrinsic parallel diffusivity

was set to 1.7 x 10^{-9} $m^2 \cdot s^{-1}$ in the estimation procedure. From the estimated model extra-cellular diffusion tensors were reconstructed allowing us to extract the traditional diffusion tensor image (DTI) measures such as the fractional anisotropy (FA) and mean diffusivity (MD). The NODDI model itself offers neurite density, orientation dispersion and free-water fraction maps.

Unbiased study-specific coordinate system

Unbiased global template space was estimated as shown in Figure 1. While estimating an unbiased atlas (coordinate system) is well investigated in cross-sectional imaging studies, there are fewer validation studies using a longitudinal design. The additional bias which we must restrict in a longitudinal study is the interpolation asymmetry that can arise when selecting only *one of the time points as a temporal representative in generating the population/study level coordinate system* as described in recent works [17]. The subject-specific average that is temporally unbiased was first estimated. The subject specific averages were then used to generate an unbiased population level average template space as shown in Figure 1, where each wavy black line represents a combination of affine and non-linear diffeomorphic transformations. These transformations and the spatial averages were estimated iteratively until convergence. We employed DTI-TK which is an open source and extensively validated image registration toolbox [18, 19] for estimating the transformations using the diffusion tensors. We note that our processing offers improvement over [20, 21]. [20] also recognizes the need for avoiding subject-specific averages and so heuristically generates a common template space. [21] does *not* generate a global coordinate system and instead involves a non-study specific stereotaxic space such as ICBM-152 which can introduce additional unwanted biases [22].

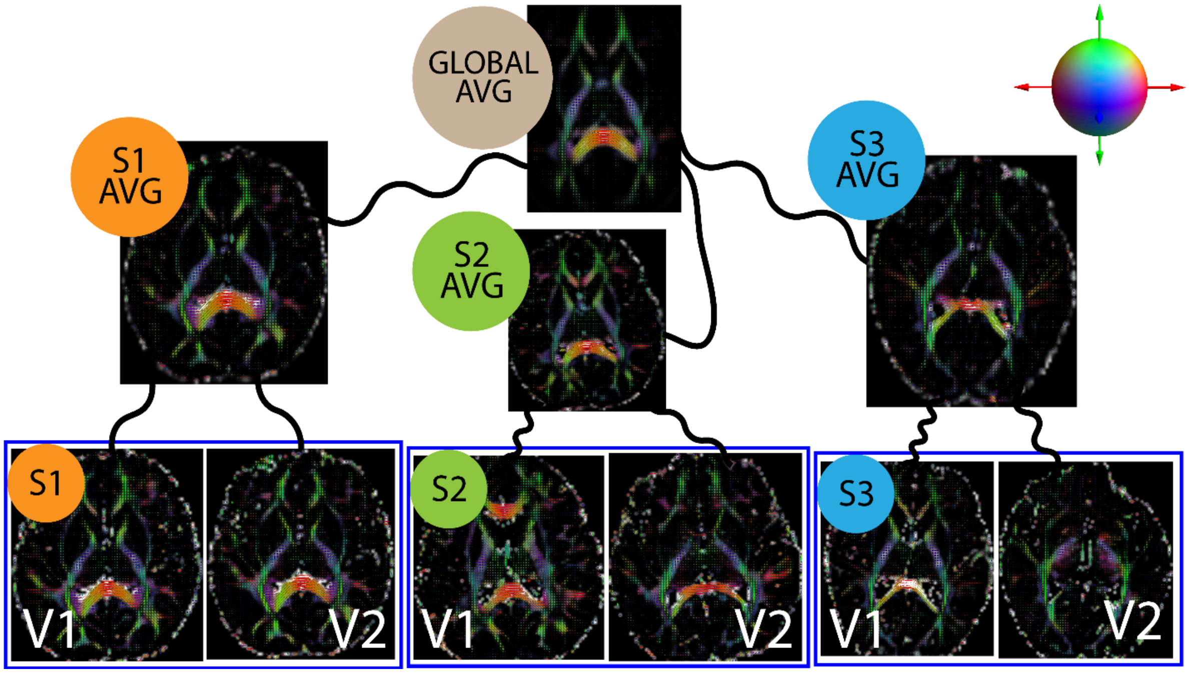

Figure S1. Unbiased estimation of the global coordinate system for the longitudinally acquired imaging data. Significance: Visits V1 and V2 are averaged first which are then used to estimate the global average. Each image represents the maps of extra-cellular diffusion tensors. These are color-coded according to their primary orientations as indicated the color-coded sphere on the top-right corner.

Once the unbiased coordinate system is estimated all the scans were transformed to this space as shown in Figure 2

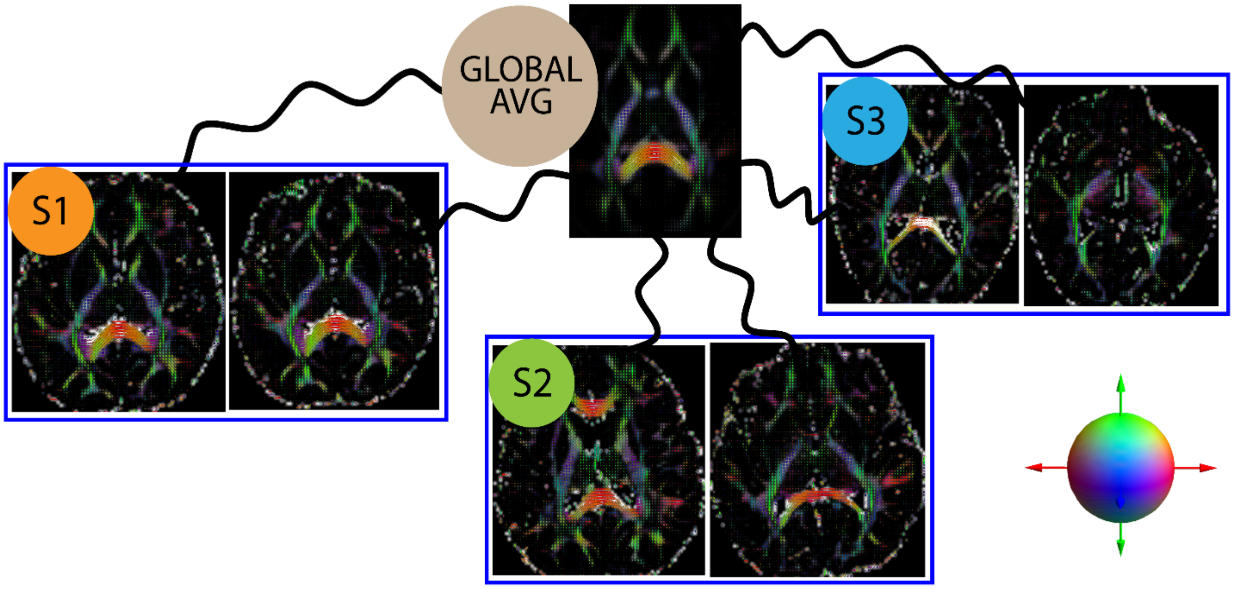

Figure S2. Registering individual scans to the unbiased template. Significance: Since the GLOBAL AVG was estimated by reducing spatio-temporal biases, the estimated transformations (wavy lines) would reduce any image processing-based confounding factors in the statistical inferences performed thereafter.

Processing for extracting regions of interest

The JHU-ICBM atlas defines 48 deep white matter regions of interest (ROIs) [23]. To obtain the diffusion measures in these ROIs, the JHU-ICBM FA template was registered to the unbiased global average (shown in Figure 1 and 2). The individual ROIs were then inverse warped into individual subject space. This is possible because the non-linear transformations

estimated using ANTS [24] are invertible up to numerical accuracy levels. Then to account for any registration imperfections the individual ROIs were thresholded on each diffusion measure map. For FA maps voxels with FA<0.2 were removed. For all the other measures first a standard deviation (σ) of the measure each ROI is estimated and all the voxels above 2σ were removed. After these refinements of the ROIs the mean diffusion measure in each of those ROIs were used as "outcome" measures in our analyses.

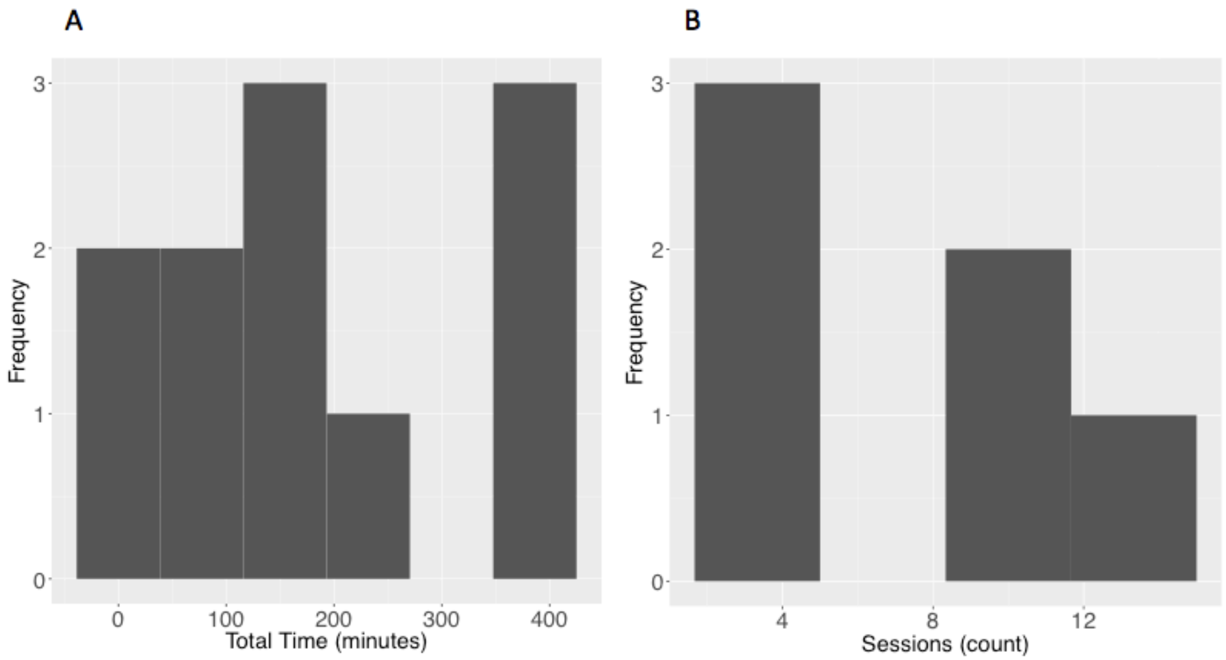

Figure S3. Compliance in Tenacity group: A timer was imbedded in the game and the recording of the time spent playing the game was automatic. No one was excluded from the analyses based on compliance. A) Participants who did not meet the requirement to play the game for at least 420 minutes (30 min x 14 days) over two-week training. B) Participants who did not meet the requirement to play the game for at least 14 sessions over a two-week training period. (A and B groups are not mutually exclusive: Most of the participants who did not play enough sessions also did not meet the total playtime requirement).

References

1. Etkin, A., Egner, T., Peraza, D. M., Kandel, E. R. & Hirsch, J. Resolving emotional conflict: a role for the rostral anterior cingulate cortex in modulating activity in the amygdala. Neuron 51(6), 871–882 (2006).
2. Kecskemeti, S. et al. MPhRAGE: A technique to simultaneously acquire hundreds of differently contrasted MPRAGE images with applications to quantitative T1mapping. Magn Reson Med 75(3), 1040–1053 (2016).
3. Cox, R. W. AFNI: software for analysis and visualization of functional magnetic resonance neuroimages. Comput Biomed Res. 29(3), 162–73 (1996).
4. Smith, S. M. et al. Advances in functional and structural MR image analysis and implementation as FSL. Neuroimage 23, S208–S219 (2004).
5. Ashburner, J. A fast diffeomorphic image registration algorithm. Neuroimage 38(1), 95–113 (2007).
6. Avants, B. B., Tustison, N. J., Song, G. & Gee, J. C. ANTS: Advanced Open-Source Normalization Tools for Neuroanatomy. Penn Image Computing and Science Laboratory (2009).
7. Woolrich, M. W. et al. Bayesian analysis of neuroimaging data in FSL. Neuorimage 45, S173–86 (2009).
8. Zhang, Y., Brady, M. & Smith, S. Segmentation of brain MR images through a hidden Markov random field model and the expectation-maximization algorithm. Med Imag, IEEE Trans. 20, 45–57 (2001).
9. Friston, K. J., Williams, S., Howard, R., Frackowiak, R. S. & Turner, R. Movement-related effects in fMRI time-series. Magn Reson Med. 35(3), 346–55 (1996).

10. Weissenbacher, A. et al. Correlations and anticorrelations in resting-state functional connectivity MRI: a quantitative comparison of preprocessing strategies. Neuroimage 47(4), 1408–16 (2009).
11. Biswal, B., Yetkin, F. Z., Haughton, V. M. & Hyde, J. S. Functional connectivity in the motor cortex of resting human brain using echo-planar mri. Mag Res Med.34, 537–541 (1995).
12. Nee, D. E., Wager, T. D. & Jonides, J. Interference resolution: Insight from a meta-analysis of neuroimaging tasks. Cogn Affect Behav Neurosci 7(1), 1–17 (2007).
13. Brefczynski-Lewis, J. A., Lutz, A., Schaefer, H. S., Levinson, D. B. & Davidson, R. J. Neural correlates of attentional expertise in long-term meditation practitioners. Proc Natl Acad Sci USA 104(27), 11483–11488 (2007).
14. Fan, J., McCandliss, B. D., Fossella, J., Flombaum, J. I. & Posner, M. I. The activation of attentional networks. Neuroimage 26, 471–479 (2005).
15. Jenkinson, M., Beckmann, C. F., Behrens, T. E., Woolrich, M. W. & Smith, S. M. FSL. Neuroimage 62, 782–790 (2012).
16. Zhang, H., Schneider, T., Wheeler-Kingshott, C. A. & Alexander, D. C. NODDI: practical in vivo neurite orientation dispersion and density imaging of the human brain. Neuroimage 61, 1000–1016 (2012).
17. Keihaninejad, S. et al. An unbiased longitudinal analysis framework for tracking white matter changes using diffusion tensor imaging with application to Alzheimer's disease. Neuroimage 72, 153–163 (2013).
18. Wang, Y. et al. DTI registration in atlas based fiber analysis of infantile Krabbe disease. Neuroimage 55(4), 1577–1586 (2011).
19. Adluru, N. et al. A diffusion tensor brain template for rhesus macaques. Neuroimage 59(1), 306–318 (2012).

20. Wu, G., Wang, Q. & Shen, D. & Initiative AsDN Registration of longitudinal brain image sequences with implicit template and spatial–temporal heuristics. Neuroimage 59(1), 404–421 (2012).

21. Guizard, N. et al. Spatio-temporal regularization for longitudinal registration to subject-specific 3d template. PloS 10(8), e0133352 (2015).

22. Fonov, V. et al. Unbiased average age-appropriate atlases for pediatric studies. Neuroimage 54(1), 313–327 (2011).

23. Mori, S., Wakana, S., VanZijl, P. C. & Nagae-Poetscher, L. MRI atlas of human white matter. Am Soc Neuroradiology, 16 (2005).

24. Avants, B. B., Epstein, C. L., Grossman, M. & Gee, J. C. Symmetric diffeomorphic image registration with cross-correlation: evaluating automated labeling of elderly and neurodegenerative brain. Med image anal 12(1), 26–41 (2008).
